# Structure of African Swine Fever Virus and Associated Molecular Mechanisms Underlying Infection and Immunosuppression: A Review

**DOI:** 10.3389/fimmu.2021.715582

**Published:** 2021-09-06

**Authors:** Yue Wang, Weifang Kang, Wenping Yang, Jing Zhang, Dan Li, Haixue Zheng

**Affiliations:** State Key Laboratory of Veterinary Etiological Biology and OIE/National Foot and Mouth Disease Reference Laboratory, Lanzhou Veterinary Research Institute, Chinese Academy of Agricultural Sciences, Lanzhou, China

**Keywords:** African swine fever virus, immunosuppression, nucleocytoplasmic large DNA virus, infection, animal disease

## Abstract

African swine fever (ASF) is an acute, highly contagious, and deadly infectious disease. The mortality rate of the most acute and acute ASF infection is almost 100%. The World Organization for Animal Health [Office International des épizooties (OIE)] lists it as a legally reported animal disease and China lists it as class I animal epidemic. Since the first diagnosed ASF case in China on August 3, 2018, it has caused huge economic losses to animal husbandry. ASF is caused by the African swine fever virus (ASFV), which is the only member of Asfarviridae family. ASFV is and the only insect-borne DNA virus belonging to the Nucleocytoplasmic Large DNA Viruses (NCLDV) family with an icosahedral structure and an envelope. Till date, there are still no effective vaccines or antiviral drugs for the prevention or treatment of ASF. The complex viral genome and its sophisticated ability to regulate the host immune response may be the reason for the difficulty in developing an effective vaccine. This review summarizes the recent findings on ASFV structure, the molecular mechanism of ASFV infection and immunosuppression, and ASFV-encoded proteins to provide comprehensive proteomic information for basic research on ASFV. In addition, it also analyzes the results of previous studies and speculations on the molecular mechanism of ASFV infection, which aids the study of the mechanism of clinical pathological phenomena, and provides a possible direction for an intensive study of ASFV infection mechanism. By summarizing the findings on molecular mechanism of ASFV- regulated host cell immune response, this review provides orientations and ideas for fundamental research on ASFV and provides a theoretical basis for the development of protective vaccines against ASFV.

## Introduction

African swine fever virus (ASFV) is a 200 nm diameter icosahedral DNA virus comprising envelope, capsid, inner capsule membrane, core shell, and inner core. The viral genome is a linear 170–190 kb long double-stranded DNA molecule with covalently closed ends. The size of the DNA is 170kb-190kb depending on the virus strain and encodes 150-200 viral proteins, including 68 structural proteins and more than 100 non-structural proteins (1–4). The repeat and loss of certain sequences in the ASFV genome is one of the factors for differences in ASFV strains from different sources or different generations of the same strain (5). P72 is the major capsid protein, which is used for serotyping of ASFV strains because of its conservativeness (6, 7). Monocyte–macrophages are the main target cells of ASFV (7). The molecular mechanism of ASFV infection in host cells is still unclear. The clathrin-dependent endocytic pathway and the macropinocytosis pathway are the probable pathways for ASFV invasion. However, the cell membrane receptors and viral proteins mediating this process remain unknown. The internalized virus particles rely on the of the host cell endosomal system to move from the edge of the membrane to the center. With the gradual acidification of the endosome, ASFV removes the outer shell and inner envelope, releasing the viral genome into the cytoplasm (8). There are two stages of ASFV replication. The first stage of replication takes place briefly in the nucleus and then a large number of DNA fragments are synthesized in the virus factory (VF) in the perinuclear region (9). Viral gene expression is divided into four stages: immediate-early, early, middle and late (10). ASFV has a set of host-independent replication and transcription mechanisms, but the translation process remains host-dependent (2, 11). Genome replication and transcription rely on many ASFV genome-encoded related proteins (5). ASFV also performs post-transcriptional and post-translational modifications, such as 5’-mRNA capping of mRNA and protein acetylation of proteins, which are favorable for intracellular viral genome expression (12, 13). The synthesized virus proteins assemble at near the VF, holding the endosomal system to transport the progeny virus to cell membrane, and then bud and release.

The viruses are identified and attacked by the host immune system. For the benefit of reproduction, the viruses have evolved several mechanisms to evade and suppress immune response. Innate immunity is the first line of defense, which is also one of the targets of viral immunosuppression. ASFV is mainly recognized by cGAS, which then transmits signals downstream by signaling pathways to produce an antiviral response. Thus, ASFV weakens the immune response by blocking signal delivery with multiple molecules on the antagonized cGAS/STING pathway (14–16). In addition, to a certain extent, cells inhibit virus spread by initiating their apoptosis procedures. Accordingly, ASFV encodes various viral proteins that inhibit both the exogenous and endogenous apoptosis pathways in the early stage (14). ASFV primarily regulates cytokines such as interferon (IFN) and tumor necrosis factor alpha (TNF-α). ASFV not only restrains IFN and TNF-α activation, transcription and expression, but also inhibits the expression of their downstream proteins (16). In addition, ASFV inhibits adaptive immune responses, such as major histocompatibility complex (MHC)-I/II expression and cytotoxic T-cell activation (14, 16, 17). The transcription factor nuclear factor kappa beta (NF-κB) induces proinflammatory factor expression. Therefore, ASFV can regulate inflammatory response by regulating NF-κB.

## Structure

**Figure 1:**
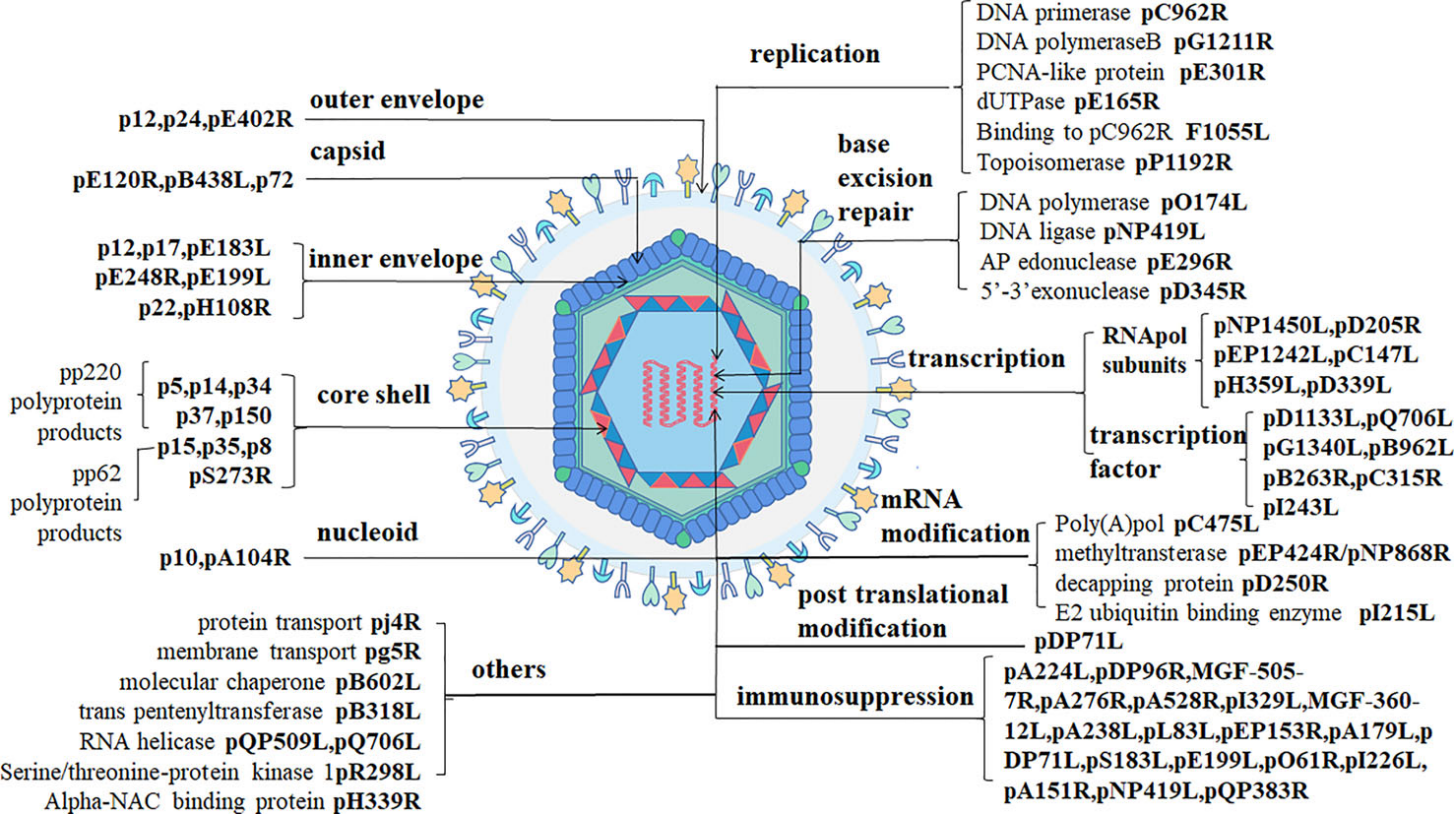
ASFV structure (1). ASFV-encoded structural proteins constitute the envelope (p12/p24/pE402R), the capsid (pE120R/pB438L/p72), and the inner envelope (p12/p17/pE183L/pE248R/pH108R/pE199L/p22). p5, p14, p34, p37, and p150 are pp220 hydrolysates produced by pS273R. p15, p35, and p8 are p62 hydrolysates. DNA-binding proteins include pA104R and p10. ASFV-encoded proteins with clear functions include those involved in viral DNA replication (pC962R/pG1211R/pE301R/pE165RF1055L/pP1192R), base excision repair (pO174L/pNP419L/pE296R/pD345R), transcription (pNP1450L/pD205R/pEP1242L/pC147L/pH359L/pD339L/pD1133L/pQ706L/pG1340L/pB962L/pB263R/pC315R/pI243L), mRNA modification (pC475L/pEP424R/pNP868R/pD250R/pI215L), post-translational modification (pDP71L), immunosuppression (pA224L/pDP96R/MGF-505-7R/pA276R/pA528R/pI329L/MGF-360-12L/pA238L/pL83L/pEP153R/pA179L/pDP71L/pS183L/pE199L/pO61R/pI226L/pA151R/pNP419L/pQP383R), and other proteins (pj4R/pg5R/pB602L/pB318L/pQP509L/pQ706LpR298l/pH339R).

ASFV is a giant icosahedral DNA virus. The diameter of the virus particles is between 260-300 nm. ASFV has a multiple-layer structure, including the envelope, capsid, inner envelope, core shell and nucleoid from outside to inside (18, 19) as shown in **Figure 1**.

### The Outer Envelope (p12/pE402R)

The outer envelope is the outermost layer of ASFV which is acquired it from the host cellular membrane during budding. Some fractions of protein pEP402R (CD2v) have been detected on the outer layer of budding virions. The viral pE402R homolog is the only marker molecule of the outside-virus structure (1). p12 promotes the adsorption of virus particles on host cells as an outer envelope protein by binding to specific receptors on the host cell membrane to mediate ASFV entry. However, other studies have shown that p12 is localized on the inner envelope of the virus using immunoelectron microscopy (1, 20).

### The Capsid (pE120R/pB438L/P72)

The diameter of the largest ASFV capsid is approximately 250 nm. The capsid components are 2,760 pseudo-hexameric capsomers and 12 pentameric capsomers. Every three p72 protein molecules adopting a double jelly-roll structure forms one pseudo-hexameric capsomer, and another five penton proteins can construct a pentameric capsomer (19). Protein pB438L is necessary for the capsid to form its vertices. In addition to proteins p72 and pB438L, pE120R also belongs to the virus capsid (1).

### The Inner Envelope (p12/p17/pE183L/pE248R/pH108R/pE199L/P22)

The third layer, the inner envelope, is a 70-Å thick lipid bilayer membrane divided from the endoplasmic reticulum (ER) (2). A previous study has pointed out that pE183R is a key protein involved in inner envelope formation. A recent study has reported the presence of p17, pE183L, p12, pE248R, and pH108R in the inner envelope using immunoelectron microscopy. p17 and pE183L primarily help to assemble the capsid layer, while p12, pE248R, and pE199L are involved in virus entry. pE248R, and pE199L are believed to be a part of the virus integration mechanism. In addition, some researchers have suggested that the p22 protein is also a component of the inner viral membrane (1).

### The Core Shell (p5/p14/p34/p37/p150/p15/p35/p8/pS273R)

The fourth layer is a 180-nm-diameter, called the core shell. Two kinds of virus polyprotein precursors, pp220 and pp62, are broken down into many mature products through viral protease (pS273R) to form the core shell. P150, p37, p34, p14 and p5 are developed from pp220, while p35, p15, and p8 are developed from pp62 (1, 2).

### The Nucleoid (p10/pA104R)

The innermost of virus particles are nucleoids. ASFV genome is approximately 170–194 kbp linear double-stranded DNA and encodes 150–170 open reading frames (10). The terminal of the viral genome consists of covalently cross-linked hairpin loops (2). p10 and pA104R are DNA binding proteins that have been detected in the viral nucleoid (1). Besides, the ASFV genome codes many non-structural proteins related to virus replication, transcription, and immunosuppression (**Figure 1**).

## ASFV Infection Mechanisms

To infect host cells successfully, viruses must undergo six events including adsorption, penetration, uncoating, biosynthesis, packaging, and shedding. ASFV is internalized within 30mip after binding to the host receptor or through macropinocytosis pathway. Virion gets into early endosomes during 1-30 minutes post-infection (mpi) and is transported into late endosomes between 30 to 90 mpi. After that, ASFV completed the shelling and genome release in the late endosomes. Gene expression can be divided into three stages: early, intermediate and late gene expression. Early genes expression occurs at 4-6 hours post-infection (hpi) and mainly encode ASFV replication related proteins, followed by the replication of ASFV genome at 6-8 hpi. Genome replication occurs first in the nucleus, mainly in the cytoplasm. After replication, the intermediate and late genes which encode virus particle structure related proteins start expression at 8-16 hpi. At 16-24 hpi, virus particles are assembling at virus factory. About 24 hpi, the assembled virus particles sprout from the cell membrane to release outside (7).

This study describes the mechanism of ASFV infection through ASFV entry, transport, genome duplication, transcription, translation, VF formation, and offspring-virion assembly and release. Monocyte-macrophages are the main host cells of ASFV. Moreover, ASFV also infects secondary target cells such as vascular endothelial cells, hepatocytes or epithelial cells (21).

### ASFV Invasion Mechanism

**Figure 2:**
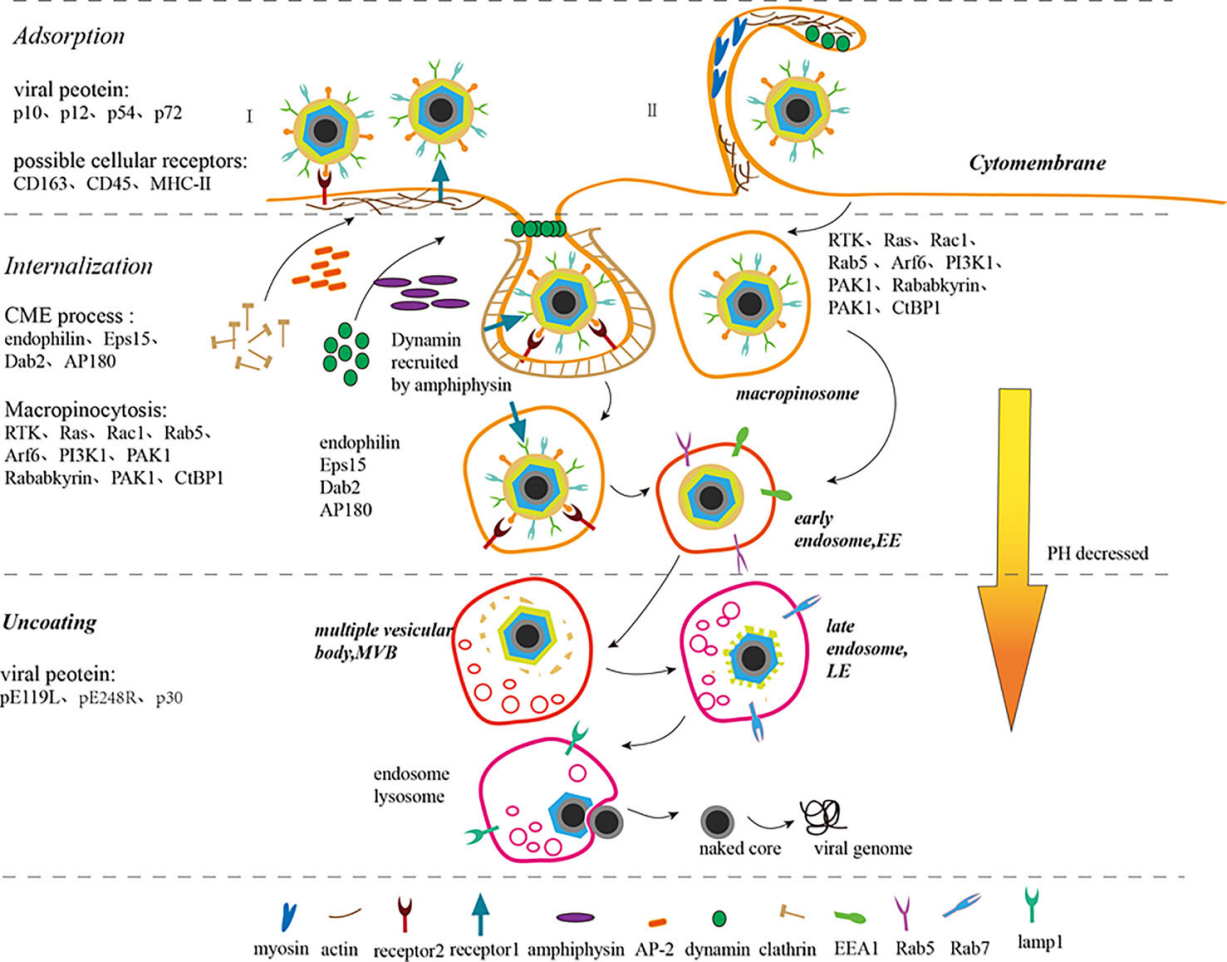
ASFV adsorption, internalization, uncoating, and release process (7–9, 21–24). ASFV enters the host cell through endocytosis (I) and macropinocytosis (II). In endocytosis (I), the virus binds to the cell receptors to form coated pits. Adaptor protein-2 (AP-2) recruits clathrin to accumulate and assemble in coated pits and amphiphysin recruits dynamin simultaneously. Disabled protein 2 (Dab2), AP180, and Eps15-interacting protein (EPsin) can also recruit clathrin. The coated pits sink downward under the action of dynamin, endophilin, and actin. Simultaneously, dynamins assemble on the depressed neck and then cut the depressed corpuscles to separate it from the cell membrane. The separated small vesicles lose clathrin, thus forming early endosomes (EE). In micropinocytosis (II), the virions induce and activate RTK, Ras, Rac1, Rab5, Arf6, and PI3K1 signals, which activate actin, thus rearranging actin and microfilaments to form ruffles or blebs on the cell membrane surface. PAK1 and Arf6 regulate the dynamic cytoskeletal changes and induce membrane bending. Rab5, Rababkyrin, PAK1 and CtBP1 induce ruffle closure. Myosin provides contractile activity for ruffle closure. The macropinosomes are equivalent to the EEs of the cell. EEs migrate, mature and are acidified within the cell to form multiple vesicular bodies (MVBs), late endosome (LE), and endosome lysosome gradually. ASFV sheds its capsid in LE, fuses its inner capsule with the restricted endosome membrane, releases its core shell into the cytoplasm, and then releases its DNA.

Previous studies have shown that ASFV entry into host cells, a receptor-mediated endocytosis process, is temperature-, energy-, cholesterol-, and low-pH-dependent. Recent studies have described this process to be clathrin-dependent endocytosis (CME) and macropinocytosis. Both CME and macropinocytosis require dynamin. The obvious negative effect of inhibition key proteins needed for ASFV entry by several pharmacological inhibitors and specific proteins indicates that the main pathway for entry is macropinocytosis. Macropinocytosis depends on actin rearrangement and also involves the cholesterol, sodium/proton exchanger (Na/H), EGFR, PKC, phosphorylated PI3K, Pak1 kinase, and small Rho-GTPase Rac1 activation (2, 21) (**Figure 2**). However, in some studies, purified viral particles did not cause significant macropinocytosis events in Vero cells and macrophages (25). Interestingly, clinical symptoms suggest that ASFV-infected macrophages show antibody-dependent enhancement (ADE). In other words, the Fc receptor may participate in this process, but further studies needed to verify this hypothesis (7). Taken together, it is easy to speculate that ASFV prefers utilizing different pathways to improve its ability to infect various target cells and adapt to the changing conditions of the infection process.

#### Adsorption

CD163 antibody inhibits ASFV infection in macrophages, which indicates that the CD163 receptor on the surface of macrophages is closely related to ASFV susceptibility. Nevertheless, the mortality, pathological anatomy, and viremia of ASFV-infected CD163 gene knockout pigs were not significantly different from those in the control pigs. This result suggests that CD163 alone is not enough for virus particles to infect host cells and other proteins are also involved in this process (9). In addition, CD45 and MHC-II play a role in ASFV invasion of macrophages (26). In short, the cell receptors mediating ASFV adsorption on host cell surface are still unknown.

Till date, studies have shown that p10, p12, p54, and p72 mediate ASFV adsorption. p10 not only binds to single-stranded and double-stranded DNA, but also enters the nucleus (8, 27). p54 and p72 neutralizing antibodies can blocks the ASFV adsorption to macrophages, indicating that p54 and p72 proteins are involved in ASFV adsorption (7). The ASFV EP402R protein mediates adsorption on red blood cell surface harboring a certain similarity with CD2v located on the cell surface (28).

#### Internalization

In the CME process, virions bind to specific receptors firstly on the cell membrane, and then form coated pits at specific sites. Adaptor protein-2 (AP-2) specifies the clathrin assembly site on the lipid membrane and promotes clathrin aggregation and assembly on the plasma membrane. Disabled protein 2 (Dab2) also exhibits clathrin assembly activity. Both AP180 and Eps15-interacting protein (Epsin) accelerate clathrin assembly *in vitro*. Dynamin binds to GDT in the presence of amphiphysin and locates on the cell membrane through interaction with PtdIns(4,5)P2 *via* its PH domain. The coated pits on the plasma membrane invaginate under the action of dynamin, endophilin and actin, and then the clathrin-formed sunken cell membrane encloses the virus particles together with cell receptors. The GDT/GTP exchange allows dynamin to fall off the coated pits and concentrate in the depressed neck. Then, the depressed corpuscles are cut by dynamin to separate them from the cell membrane. The isolated vesicles lose clathrin and form early endosomes (EE) (8, 22). Related studies have shown that clathrin-coated pit formation will be inhibited in Eps15-deficient Vero or WSL cells. Cholesterol is also essential for clathrin-coated vesicle formation (29).

Macropinocytosis is a non-selective endocytosis process. During macropinocytosis, virus particles induce RTK and Ras activity which then activate downstream molecules, such as Rac1, Rab5, Arf6, and PI3K1, causing actin and microfilaments rearrangement to form ruffles or blebs on the cell membrane surface. While virions are adsorbed on the cell membrane surface, PAK1 regulates the dynamic changes of the cytoskeleton and Arf6 induces membrane curvature to wrap the virus particles in the raised folds. Subsequently, Rab5 with its effector molecules Rababkyrin, PAK1 and CtBP1 induce ruffle closure, while simultaneously providing contractile activity for the closure of endocytic vesicles. The formation of macropinosomes, corresponding to early endosomes (EEs), shows that the virus has successfully completed internalization (8, 23). Viruses that enter cells through this process include vaccinia virus, adenovirus, and picornavirus. The outer envelope of mature virus particles is rich in phosphatidylserine, which is a phospholipid necessary for cells to absorb cell debris through macropinocytosis. Changing the lipid content of the outer membrane of the virus can result in failure of the internalization into cells, which provides a strong evidence that the virus particles induce the macropinocytosis in cells by simulating apoptosis (23). However, some researchers believe that ASFV does not specifically activate the process of micropinocytosis because macropinocytosis can also be produced by the intake of many nutrients and removal of apoptotic cell debris (8).

The two methods of viral internalization are not antagonistic, but cooperate with each other, for instance, actin rearrangement is also involved in the CME process.

#### Uncoating

EE migrates and matures within the cell, transforming into acidic multiple vesicular bodies (MVBs), late endosomes (LEs), or endosomal lysosomes fused with lysosomes. In LE, the ASFV completely uncoats in an acidic environment. The uncoating of the virus requires acidic conditions, and shelling is inhibited if acidification of endosomes is prevented (8). Factors affecting virus uncapsidation also include the cholesterol entry through the endocytic pathway and the regulation of the ubiquitin-proteasome of host cells. Inhibiting the ubiquitin-proteasome pathway prevents ASFV decapsidation, suggesting that ubiquitin-proteasome is also essential for ASFV uncoating. The ubiquitin-proteasome system is required for ASFV replication (30, 31). Then, the inner envelope membrane fuses with the restricted endosome membrane and the viral DNA is set free from the core shell following the release of the core shell into the cytoplasm (8). Vaccina virus (VACV), which also belongs to the NCLDV family, releases virions from endosomes to the cytoplasm through an entry fusion complex (EFC) composed of 11 viral polypeptides. Interestingly, the ASFV-encoded protein pE199L encoded by ASFV is thought to be essential for fusion of the inner membrane with the restrictive endosome membrane and the release of the core shell because of its sequence similarity with the components of VACV EFC including G9, A16, and J5 proteins (32). The sequence and structural characteristics of pE248R, also located in the inner capsule membrane of ASFV, are identical to those of the L1 protein, which is also one of the compositions of EFC. Therefore, it is supposed that ASFV may have a fusion mechanism composed of pE199L and pE248R, which is a simplified version of the VACV EFC. Homologous EFCs have also been found in other viruses in the NCLDV family, such as iridoviruses and phycodnaviruses (21, 32). Furthermore, another study has reported that the protein pE248R located in the inner envelope membrane mediates the fusion of the viral inner membrane and the restricted endosome membrane with the cholesterol present in the endosomal membrane. This phenomenon validates the speculation that ASFV has a similar structure of EFC. In addition, the process of ASFV DNA release from core shell to the cytoplasm is supposed to be similar to that of other viruses depending on the host cell’s ubiquitin-proteasome pathway (21). Other studies have shown that the release of virus particles is also impeded by microtubule inhibition indicating that microtubules play an important role in ASFV invasion (8). The structural protein P30, an early protein encoded by ASFV, is also believed to play a significant role in virus internalization (7).

### ASFV Traffic Mechanism in Host Cells

The transport of virions from the EE to the VF and then the cell membrane depends on the function of microtubules (33). After the virion is internalized, the vesicles successively form early endosome (EE), mature endosome (ME), and late endosome (LE) in the cell (24). As EEs containing ASFV mature into LEs, the endosome cavity acidizes steadily, the molecular markers on the endosomal membrane surface gradually change, and finally the virus particles are unshelled in an acid environment. ASFV infection redistributes the entire vesicle system so that virus particles can be easily transported to the perinuclear region (3). Immediately after infection (5-30 mpi), capsid and inner envelope proteins co-localize with specific markers such as EEA1 and Rab5 in EE and macropinocytosome. In the late-stage (30-90 mpi), the viral inner envelope protein (P17) or core shell protein (P150) co-localize with the late endosomal marker (Rab7), a key regulator of ASFV infection, and the lysosome marker (Lamp1) (21, 33). After the virus particles are assembled in the VF, the kinesin uses a multi-terminal microtubule motor to transport the “cargo” from the VF to the cell membrane.

Intriguingly, ASFV may also increase microtubule stability by over-acetylating microtubules to ensure that mature virus particles are transported to the plasma membrane. The actin cytoskeleton is considered to be a possible alternative route for retrograde viral transport (34).

### ASFV Replication and Gene Expression Mechanism in Host Cells

**Figure 3:**
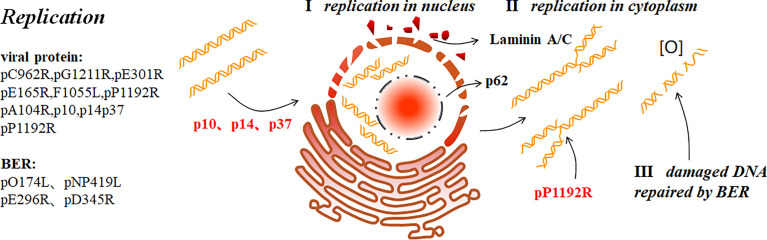
ASFV replication (9). ASFV first replicates briefly in the nucleus (I), and then the cytoplasm (II). p37 mediates the nucleocytoplasmic transport of ASFV genome. In addition, p10 and p14 can also enter the nucleus. ASFV replication in the nucleus ruptures the nucleolus and nuclear membrane, thus collapsing laminin A/C and recruiting the nucleoporin p62 to the periphery. ASFV produces short DNA fragments the nucleus and long fragments in the cytoplasm. pP1192R catalyzes transient nick generation in double-stranded DNA and promotes the translation process. To prevent active oxygen-mediated damaged DNA from interfering with replication, ASFV also uses BER to repair the damaged DNA (III).

**Figure 4:**
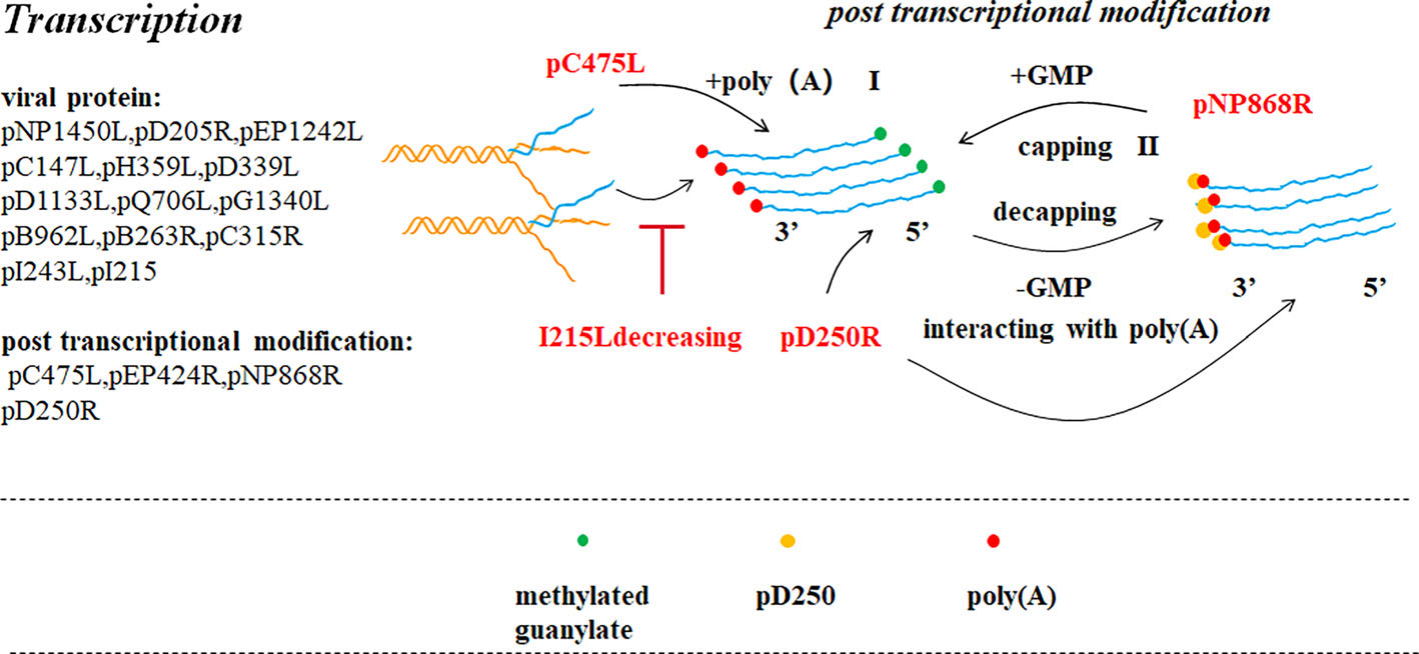
ASFV transcription and post-transcriptional modification (39). Pre-mRNA obtained after ASFV transcription is modified in the cytoplasm by capping the 5’ end (II) and poly adenylating the 3’ end (I). ASFV encodes a polyadenosine enzyme (pC475L) that may be involved in 3’ end modification. ASFV pNP868R participates in 5’ end modification. Ba71V D250R encodes a decapping protein (ASFV-DP), which could interact with poly(A) of the 3’ end.

**Figure 5:**
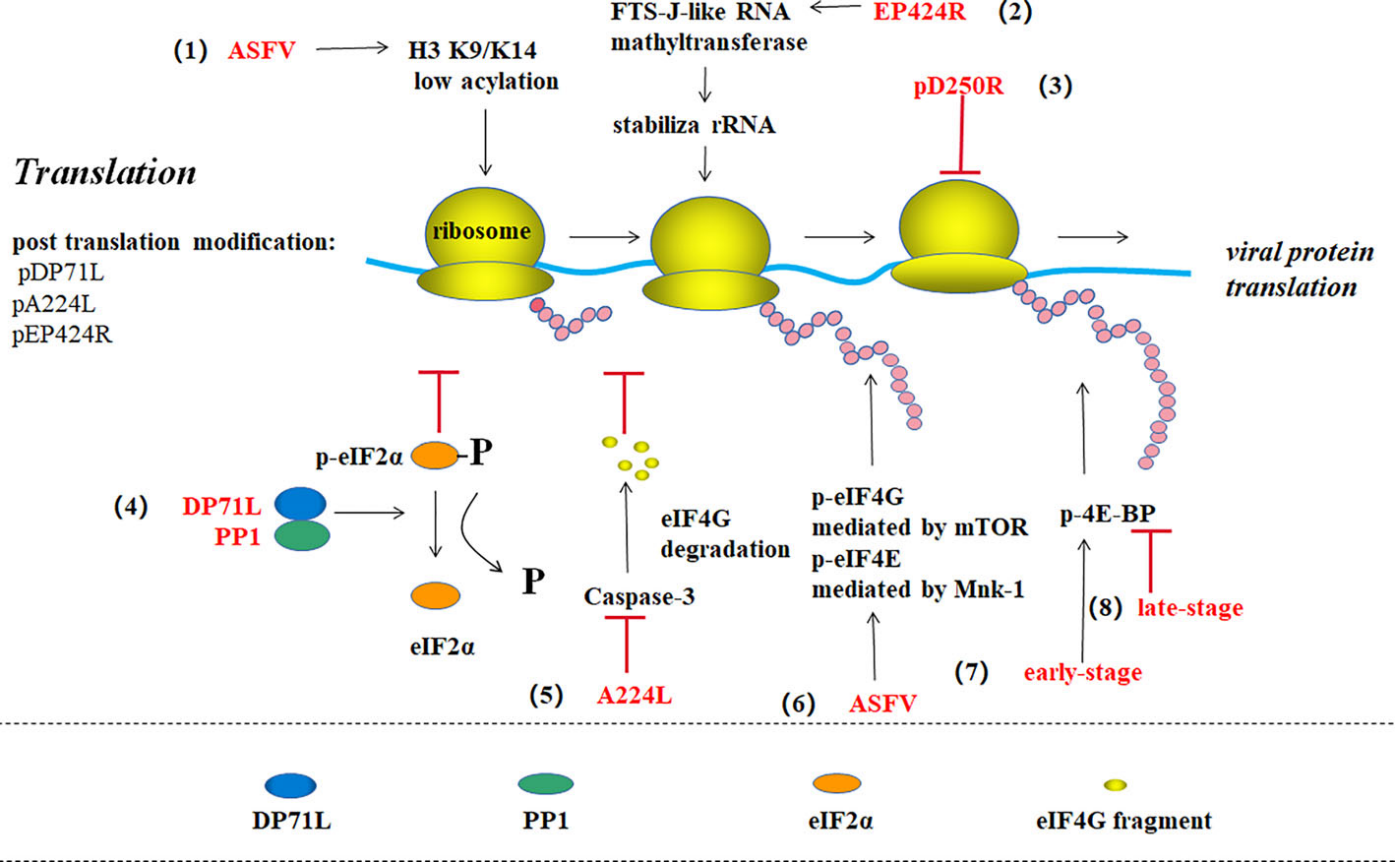
ASFV translation and post-translational modification (39). (1) ASFV promotes viral gene expression by maintaining the host histone H3 K9/K14 in a low acylation state. (2) EP424R-encoded FTS-J-like RNA methyl transferase may stabilize rRNA in cells and prevent protein synthesis shut down. (3) pD250R inhibits viral translation by reducing the number of transcripts. (4) The virus protein DP71L binds to host phosphatase 1 (PP1) to form eIF2 alpha dephosphorylation, thus enhancing viral protein synthesis. (5) ASFV IAP homologous protein A224L inhibits the Caspase-3-mediated eIF4G degradation to promote translation. (6) ASFV promotes mTOR-mediated eIF4G phosphorylation and Mnk-1-mediated eIF4E phosphorylation to enhance protein synthesis. (7) ASFV promotes 4E-BP phosphorylation and viral protein translation in the early stage of infection, while (8) promotes 4E-BP dephosphorylation in the late stage to inhibit translation.

ASFV, released from the endosome, migrate to the perinuclear microtubule tissue through activating the microtubule system by the viral inner envelope protein pE183L (p54) combined with the microtubule dynein for the DNA replication and biosynthesis (9, 35, 36) (**Figures 3–5**). Viral gene expression is divided into four stages: immediate, early, intermediate, and late stage. Early genes are expressed within 4-6 h of virus infection, including proteins and enzymes necessary for viral genome replication, required for late gene expression and multi-gene families related to immune evasion. After ASFV infection for approximately 6-8 h, the virus begins to replicate. Although replication of ASFV mainly occurs in the VF in the cytoplasm, there is also a brief moment in the nucleus for duplication in the early stage. After 8-16 h, mid- and late-stage genes begin to express to synthesize structural proteins required to form nascent virus and early transcription factors packaged in the virus particles (7, 37). Studies have shown that there are 68 types of viral proteins through mass spectrometry analysis, including structural proteins and non-structural, which are indispensable for virus transcription, DNA repair, and protein modification (38).

#### Transcription

ASFV encodes POLV subunits and the TATA-binding protein (TBP)-like protein (B263R). ASFV C315R encodes a TFIIB-like factor, and I243L encodes a transcription splicing/elongation factor TFIIS homolog (37, 40). Based on the above studies, we can assume that ASFV has a host-independent transcription system which is similar to the eukaryotic POLt transcription mechanism. In the POLII transcription mechanism in eukaryotic cells, TBP binds to the TATA box, and then TFIIB binds to the TFIIB recognition element (BRE). Finally, RNA Pol and related general transcription factors (GTFs) are recruited to form the pre-transcription initiation complex (PIC). The energy generated by ATP hydrolysis promotes transcription initiation. Factors promoting transcription elongation include TFIIS and SPT5. Hence, it has been suggested that ASFV can also forms a similar PIC. Recent studies have shown through sequencing that TA* and TA*TA motifs exist at the initiator (Inr) upstream of the TSS in the early and late promoter sequences respectively, and a conservative early promoter motif (EPM) and late promoter motif (LPM) have been found in the region further upstream of the TSS. The EPM is similar to the upstream control element (UCE) of the VACV. UCE binds to the transcription initiation factor heterodimer composed of D6 and D7. VACV and ASFV both belong to the NCLDV family. Therefore, from the transcription mechanism of VACV, it can be inferred that the ASFV- encoded D6/D7-like factor can binds to the EPM upstream of the early gene, inducing early promoter transcription. The virus-encoded TBP/TFIIB homolog expressed in the early stage of infection, is recruited to the LPM upstream of the late gene and promotes late or post-replication transcription. However, apart from the same T/A-rich nature and its location at the upstream of the TSS, LPM has no obvious similarity to the TATA box (37). Through TSS analysis, it has been found that ASFV increases protein diversity and retention by using alternative TSS, thereby synthesizing mRNA shortened at the 5’-terminal and encoding N-terminal truncated proteins. Some studies have also shown that the transcription mechanisms of ASFV and VACV are host -independent and similar to the transcription of certain yeast plasmid-encoded toxin genes. These results suggest that the NCLDV family and the killer plasmids share a common evolutionary pathway (37). While studying ASFV transcription termination by the third NGS, a polyU termination motif has been detected in the RNA of approximately 2/3 of early and late genes, but not in nearly 1/3 of the RNA. Therefore, ASFV transcription termination may be factor-dependent combined with the conservative evolution of VACV RNA helicase.

After transcribing, the pre-RNA undergoes 3′-polyadenylation and 5’-capping in the cytoplasm. Compared with VACV, it is conjectured that adding the PolyA tail to the 3′-terminal of the transcript involves the participation of an ASFV-encoding polyadenylation enzyme (C475L) (5). Three enzymes are involved in 5’-capping: 5’triphosphatase (TPase), guanylate transferase (GTase) and methyltransferase (MTase). ASFV PNP868R^MT^ a type I MTase folds according to its structure analysis which remained significantly similar to cap MTase in many cells and viruses. The high conservation of key residues indicates that the recognition characteristics of PNP868R^MT^ cap are similar to those of the host cell and virus particles (12). Another *in vitro* study has reported that Ba71V D250R encodes a decapping protein (ASFV-DP), which interacts with ribosomal protein L23a and the mRNA cap structure located on the ER and also interacts with poly(A) RNA through the N-terminal structure resulting in a decrease in the number of transcripts of the virus and host cells to shut off the cell and regulate the time of viral gene expression (41).

#### Translation

ASFV proteins synthesis depends on the host translation mechanism of the host cell. ASFV hijacks eukaryotic initiation factors (eIFs), including eIF2, eIF4F, eIF4G and eIF4E (41) (**Figure 5**). Moreover, ASFV I226R and I243L are expressed in multiple independent promoter-controlled periods (7). Interestingly, most post-transcriptional ASFV RNAs contains AU and AUAU-leaders (37). Whether they improve translation efficiency like 5`-RNA poly(A) leaders in eukaryotic cells and VACV is still unknown. Researchers have found that the E2 ubiquitin-conjugating enzyme (I215L) is an early expression gene. I215L expression decrease directly leads to B646L transcript downregulation. It has been speculated that I215L participates in multiple events in the ASFV life cycle based on the monoubiquitination, diubiquitination, and polyubiquitination function of I215L (3, 42, 43). Proteins related to the core shell and proteins involved in ASFV DNA replication may be targeted by ubiquitin-proteasome (3, 30). These studies indicate that viral replication and late gene expression are mediated by the ubiquitin pathway.

ASFV regulates the DNA translation pathway through multiple methods. For example, the virus protein DP71L binds to host-encoded phosphatase 1 (PP1) and dephosphorylates eIF2α to enhance viral protein synthesis (44, 45). ASFV protein A224L, an IAP homolog, also increases viral protein synthesis by inhibiting caspase-3-mediated eIF4G degradation in the eIF4F complex (eIF4A, eIF4E, and eIF4G) (46, 47). ASFV can stimulate mTOR-mediated serine108 phosphorylation on eIF4G and Mnk-1-mediated serine209 phosphorylation on eIF4E to enhance viral protein products (47). ASFV promotes 4E-BP phosphorylation to enhance viral protein translation in the early stage of infection, while promotes 4E-BP dephosphorylation to inhibit translation in the late-stage (2). ASFV also increases the quantity of translation component eIFs remaining in the viral VF, which promotes viral protein synthesis while inhibiting host protein synthesis (3). ASFV also has the ability of making histone H3 K9/K14 of host cell in a low-acylation state to promote viral gene expression (13). In addition, some researchers discovered that EP424R-encoded FTS-J-like RNA methyltransferase may stabilize rRNA in cells to prevent protein synthesis machine shutdown (5).

#### Replication

The nucleus is the starting site for ASFV DNA replication which then diverts into the cytoplasmic (7). The presence of the nucleus is essential for the early ASFV DNA synthesis (48). C962R of ASFV encodes a DNA primerase similar to that encoded by VACV (D5). This primerase may function like D5 which plays a role in DNA replication initiation and modifies the DNA replication mode. Thus, a new pattern similar to that of VACV replication initiation has been proposed: replication is initiated by introducing a single-stranded gap near one or both ends of the genome. The exposed 3`-OH acts as a primer for DNA polymerase when the DNA is synthesized from the end of the genome. This creates a “head-to-head concatemers” where the ends of the nascent and template strands are self-complementary and folded forming a self-starting hairpin structure to initiate DNA replication. The ASFV DNA fragments produced in the nucleus are short fragments that form long fragments or genomes with large molecular weights in the cytoplasm, but not in the nucleus. Next, large DNA fragments in the cytoplasm gradually develop into mature cross-linked DNA (5). p37 is involved in the nucleocytoplasmic viral DNA transport during ASFV replication. In the early stage of infection, p37 accompanies viral DNA entry and accumulation in the nucleus from the cytoplasm. After replication in the nucleus, they are transported to the cytoplasm and accumulate in the VF. Nuclear export protein 1 (CRM1) is not necessary for p37 to perform this function. Moreover, p14 also has nuclear transport activity, which is different from p37 as p14 only enters the nucleus and is not exported from the nucleus to the cytoplasm (7, 49, 50). In addition, p10 contains a functional nuclear localization signal (NLS) that can be introduced into the nucleus autonomously in yeast cells and mammalian cells; in the late-stage. p10 protein accumulates in the nucleus suggesting that p10 may perform important functions in the nucleus (51).

ASFV replication mostly depends on its own gene expression. DNA polymerase B, PCNA-like protein, nucleoside triphosphatase and protein fused with DNA primerase encoded by G1211R, E301R, C962R and F1055L respectively, participate in the initiation point replication (5, 7). In the early stage of infection, ASFV ruptures nucleolus and nuclear membrane, and phosphorylates and then degrades laminin A/C. The resulting membrane fragments are then recruited to the replication site, and nucleoporin p62 is recruited to its periphery. These phenomena may promote viral genome replication (52). In addition, ASFV can also promote H3K9me3(histone H3) and HP1β (heterochromatin protein 1 isoforms) foci formation at early stage with HP1α and HDAC2 (histone deacetylase 2) enrichment at nuclear. Therefore, ASFV was believed to encourage heterochromatinization of host genome, which may be beneficial for ASFV infection (53). Sulfite sequencing has shown that the nascent ASFV DNA in infected Vero cells is not methylated by the host proteins. As CPG methylation is one of the methods for cells to resist foreign nucleic acids, it has been revealed that ASFV may have a replication mechanism that is at least partially host-independent (11). The two ASFV-encoded RNA helicases, QP509L and Q706L, are conserved and speculated to be involved in mid- and late-stage viral gene replication and transcription with non-redundant effects, by detecting their mRNA levels. Recent research reveals that QP509L and Q706L are essential for ASFV producing infectious virus particles (54). The ASFV-encoded topoisomerase II (pP1192R) is transcribed in the early stage and involved in virus replication, transcription, genome agglutination, and separation, which catalyzes double-stranded DNA transient nicking (3, 39). However, some other researchers detected pP1192R at intermediate and late phases co-localized with VF (55). It has been found that the purified SFV ORF P1192R-encoded protein in Saccharomyces cerevisiae effectively decomposes kinetoplast DNA (kDNA) and promotes excessive relaxation of supercoiled DNA (7). It is suggested that the activity of PtdIns-converting kinases is necessary for intracellular ASFV replication site establishment, as inhibiting PIKfyve reduces ASFV replication level (33). Furthermore, previous studies have shown that histone-like proteins (pA104R) could bind to ssDNA or dsDNA and cooperate with pE199L to keep DNA-supercoiling activity. Therefore, it is easy to believe that both pA104R and pP1192R play an important role in ASFV replication (7, 32, 56, 57).To avoid the decrease in virus titer due to reactive oxygen-induced DNA damage, ASFV uses the DNA glycosylase of the host cell to repair the damaged DNA through the base excision repair (BER) pathway. The enzymes involved in this process include DNA polymerase type X (O174L), DNA ligase (NP419L), type II polypurine/cyclic nucleoside endonuclease (pE296R), PCNA-like protein (pE301R), and 5′–3′ exonuclease enzyme PD345R (5).

### ASFV VF Formation Mechanism

ASFV hijacks and changes the cellular trans-Golgi endosomal network system to facilitate viral infection. The host adaptor protein AP-1 mediates protein transport from the trans-Golgi network (TGN) to the endosome, recruits clathrin to form clathrin-coated vesicles and selectively carry cargo by recognizing protein sorting signals. AP-1 is recruited to the TNG membrane under the manipulation of small GTPase ADP-ribosylation factor 1 (Arf1). Intracellular AP-1 localization and clathrin-encapsulated body/endosome migration in the cell induces new membrane structure formation due to the interaction between the outer envelope proteins CD2v and AP-1 (31). Previous studies have reported that various forms and small membrane fragments are involved in the early stage of VF formation, which grows into curved membranes and then forms assembly intermediates. These assembly intermediates of the membrane accumulate and aggregate to form a network structure, while others have observed a heliciform structure (38, 58). Together with the viral genome, viral replicase, and host proteins, these membrane structures comprise the center of viral replication and morphogenesis called the VF and is located in the microtubule organizing center (MTOC) (31). The presence of structural proteins p54 and p34 has been observed in the VF membrane structure. Confocal microscopy has shown that p72 and p54 in the VF are morphologically different. p72 is punctate, while p54 is a polymorphic structure that may be related to the connection between p54 and newly formed virus particles (38). Interestingly, ASFV infection reshapes cholesterol distribution in the cells (31). The intake of cholesterol to cell is a key factor for virus to enter the host cell, replicate and form the VF. On the contrary, cholesterol is not allowed to accumulate in the endosome, otherwise virus particles will be retention in the endosome. A large number of lipid transfer proteins, such as oxysterol binding protein (OSBP), mediate lipid and ion exchange in the VF. Viruses utilize the membrane contact site (MCS) to ensure lipid transfer to the VF (3, 31, 59). The virus particles recruit PI4P kinase to form PI4P aggregates in large numbers, which then recruits OSBP to form a large quantity of MCSs, which accumulate cholesterol through cholesterol and PI4P exchange. In addition, the acyl-coenzyme A binding domain containing 3 (ACBD3) required to complete lipid exchange and phosphatidylinositol-phosphate-4-kinase IIIβ (PI4Kβ) has been found in the VF of ASFV-infected cells (59). Studies have shown that ASFV-encoded g5Rp has a Nudix hydrolysis motif, which may play a role in regulating virus morphogenesis involving inositol diphosphate polyphosphate-mediated membrane transport (60). The ASFV j4R protein interacts with the α-chain of nascent polypeptide-associated complex (NAC) which indicating that j4R may be involved in the transport of newly synthesized proteins to the VF (28).

The complete VF is a single structure lacking an external restriction membrane, and is surrounded by mitochondria and vimentin cage, which provides a physical scaffold for VF or prevents viral components entering the cytoplasm (33, 61). VF morphology changes during viral infection. Nevertheless, membrane formation, flow pathway to VF, host cell rearrangement mechanism of host cell due to VF formation, and the relationship between VF and host cell membrane are still unclear (38).

### Mature Virus Particle Assembly and Progeny Virus Release Mechanism

**Figure 6:**
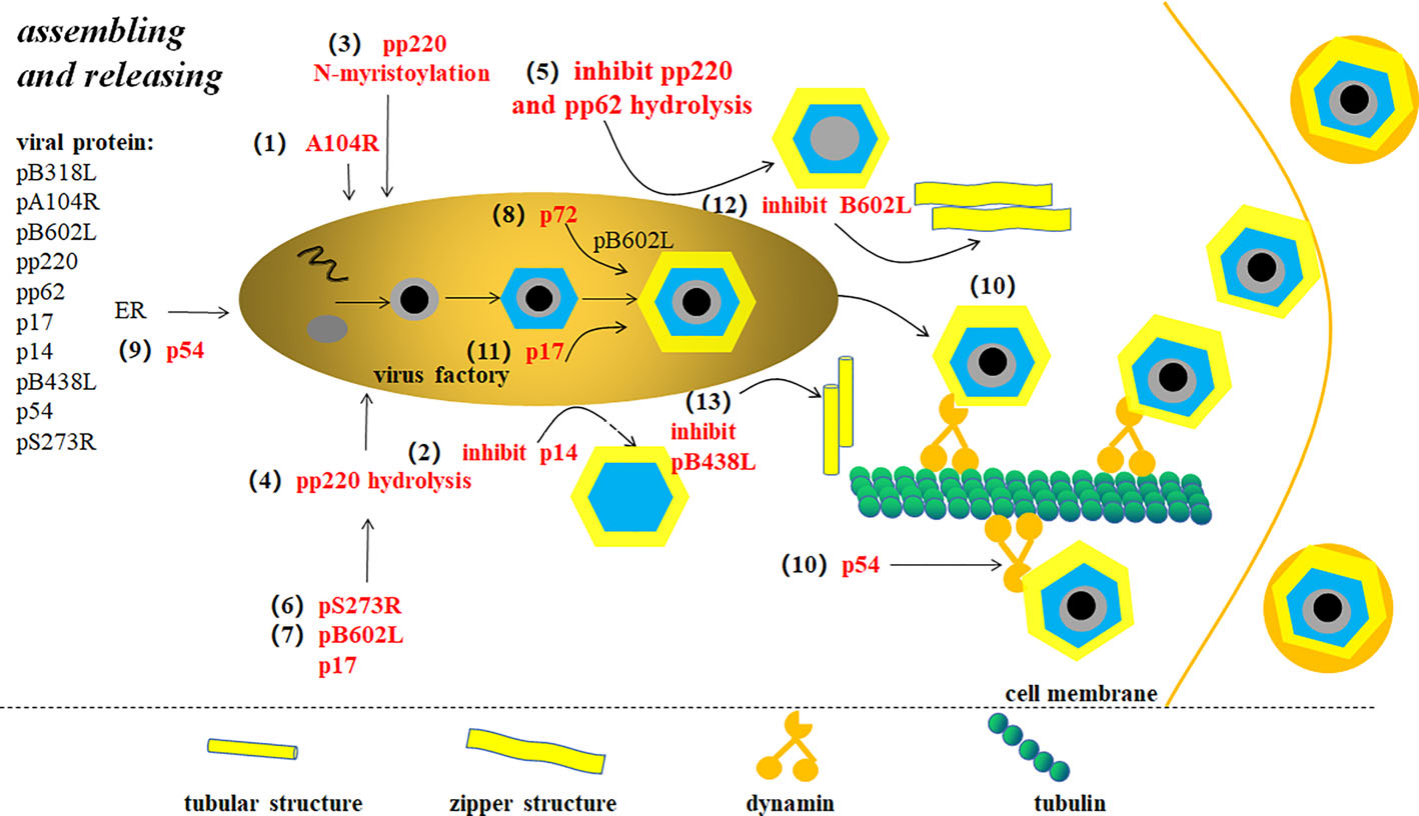
ASFV assembly and release (9). Changes in the virus particle morphology occur in virus factory (VF). (1) A104R is located in the core shell and involved in ASFV genome packaging. (2) p14 expression inhibition produces defective interference particles without a central nucleoid. (3) The core shell adsorbs on the inner membrane depending on pp220 N-bean acrylamide. (4) pp220 hydrolysis separates the core shell from the inner capsule membrane, thus concentrating, condensing, and merging the nucleus. (5) pp220 and pp62 hydrolysis inhibition leads to non-nuclear or non-infectious virus particle production. (6) ASFV proteolytic enzyme pS273R catalyzes protease processing of polyprotein precursors. (7) Proteins that affect pp220 and pp62 hydrolysis include pB602L and p17. (8) The assembly of the major capsid protein p72 requires the assistance of B602L-encoded molecular chaperone. (9) p54 expression inhibition interrupts endoplasmic reticulum (ER) transfer to virus assembly site. (10) P54 proteins interact with microtube power complexes by connecting directly to dynein. Newly-synthesized viruses are linked to kinesins, which drives the virus to move from VF to extracellular. (11) p17 plays an important role in capsule precursor conversion into an icosahedron intermediate. (12) pB602L synthesis inhibition forms abnormal “zip-like” structure instead of icosahedron. (13) Membrane protein pB438L expression inhibition forms abnormal tubular structure instead of icosahedron.

ASFV is assembled at the VF of the host perinuclear region, which is concentrated in the microtube formation center and the Goerki complex (62) (**Figure 6**). Morphological changes in viral particles occur in the VFs where the core shell proteins of virions are deposited on membrane assembly intermediates to form the core shells (38). A104R, located in the core shell, is a topological isomerase II with high affinity for double-stranded DNA, and thus has been suggested to participate in ASFV genome packing (3). However, pP1192R was thought to participate in genome segregation to facilitate the separation of newly-replicated DNA molecules. B354L and D345L are suggested to involve in processing DNA ends for strand exchange or single-strand annealing during recombination (63). p14, a late-stage non-structured protein expressed by ASFV 9GL is suppressed, leads to defective interference particles without a central nucleoid (64). The core shell is tightly attached to the inner cystic membrane at first which relies on the N-myristoylation of the viral polyprotein pp220. With viral particle maturation, the core and inner cystic membranes are separated inside the internal capsule. Simultaneously, the virus nucleoid is concentrated, condensed, and merged, which are generally considered to be inseparable from pp220 hydrolysis (21, 38). Inhibiting pp220 and pp62 hydrolysis produces non-nuclear or non-infectious viral particles. pS273R, a protein hydrolase, catalyzes the processing of proteases involved in polyprotein precursors. In addition, pB602L and p17 also affects core shell assembly to the inner membrane by affecting pp220 and pp62 hydrolysis (27). After the assembly of the internal capsule is completed, the coat proteins deposited on the membrane assembly intermediate are fitted together one by one making the membrane precursor gradually form an icosahedron structure (62). The packing of the main coat protein p72 requires the assistance of a B602L-encoded molecular chaperone (5). The transmembrane structural protein p54 is essential for ER collection and its transformation into the viral membrane precursor (65). Studies have shown that p54 is antiparallel, exposing cysteine at the N-terminal in the ER, leading to disulfide bond formation-mediated ER collapse (61). Linear array assembly of structural proteins along the ER membrane may also be a cause of ER collapse. Structural proteins assemble on the collapsed ER, forming zipper-like stacking (61). Furthermore, P54 interacts directly with the microtubule dynamic complex by connecting with dynein light chain 8 (LC8) (27). Restraining p54 expression interrupts ER transfer to the virus assembly site, which in turn prevents ER conversion into the membrane precursor, reducing the number of virus offspring (65). With regard to the assembly of icosahedron in the NLDV family, the most popular illustration is the spiral mechanism. In other words, icosahedron assembly starts at the five-fold vertices and is carried out continuously in a spiral manner. Xianjiao et al. believe that the tape measure protein (TmP) of the minor capsid protein (mcp) plays an important role in icosahedra packing. Experiments show that mcp forms a network structure under the major capsid protein (MCP) to stabilize adjacent virocapsomers. Through analysis, TmP is thought to control capsid size by connecting to the adjacent five-fold vertices and defining the boundaries of trisymmetrons. It is thought to provide a mechanism of assembly from the initial vertices to the adjacent vertices (66). p17 plays a significant role in the transformation of the capsule precursor into icosahedral intermediates. pB602L synthesis inhibition decreases p72 protein and delocalization of the shell protein pEl20R, which produces abnormal “zip-like” structure instead of the icosahedron (67). Inhibiting the expression of the membrane protein pB438L results in abnormal tube-like structures instead of an icosahedron (68). Therefore, pB602L and pB438L play an irreplaceable role in capsid formations. In addition, ASFV-coded trans-pentenyl-transferase (B318L), positioned at the virus assembly site, is associated with the viral membrane precursor from the ER, which may play a role in VF and/or virus assembly (5).

Finally, viruses are transported directly and widely using micro tubes after finishing the assembly, leaving the VF to plasma membrane (33). Mature virus particles leave the VF, pass through the cytoplasm, and are released by the host cells. The interaction between p54 protein and dynamin light chain (LC8) controls the intracellular transport of the microtubule dynamic complex. The direct binding of virus structural proteins with the small molecular dynamic complex indicates the molecular mechanism of microtubule-mediated virus transport. pEl02R and pE120R binding with the main shell protein p72 also have an essential effect on virus transport from the assembly site to the cytomembrane (69). Some studies have shown that newly synthesized viruses move to extramembranous from VF by linking them to kinesin (33).

## ASFV Immunomodulation Mechanisms

After ASFV infects porcine macrophages, the hosts first induce an innate immune response and then produce an adaptive immune response. The genome of ASFV is DNA, which can be recognized by cGAS (a kind of pattern recognition receptors (PRRs) of DNA) as pathogen-associated molecular pattern (PAMP), and then activates the expression of type I IFN through cGAs/STING signal pathway. The downstream antiviral genes of type I IFN, such as IFIT family, are also activated and expressed. Type I IFN and interferon stimulated genes (ISGs) can resist ASFV infection. Macrophages and dendritic cells are the main effector cells of natural immune response induced by ASFV infection. Macrophages enable to release a series of cytokines, such as IL-1a, IL-1b and IL-18, and express MHCI to start the adaptive immune response. Plasmacytoid DC (pDC) and natural killer (NK) cells can produce a large amount of interferon to resist ASFV infection. After 8 days of ASFV infection, the antibody level gradually increases, which suggest that there is a humoral immune response in the host. These neutralizing antibodies have a certain protective effect. In addition, cellular immunity, such as CD8α+ T cells, also plays an important antiviral role in ASFV infection. CD4 + CD8 + double positive (DP) T cells can secrete perforin and granzyme, which may also play a role in resisting ASFV infection (70).

However, to survive and produce progeny viruses, the parental virus encodes various proteins to evade the host immune response. This study summarizes the mechanism of ASFV immune evasion and immunosuppression from several aspects, such as the cGAS/STING pathway inhibition, cytokine and chemokine expression regulation, apoptosis and autophagy inhibition, adaptive immune response inhibition, and host inflammation regulation (**Table 1**).

**Table 1:** African swine fever virus (ASFV) regulates inflammation through regulating proinflammatory cytokines, inflammatory mediators, ALR and NF-κB pathway.

Regulation pathway	Viral protein	Target molecule	Effect
**proinflammatory cytokines**	pL83L (71, 72)	interact with IL-1β	**/**
**inflammatory mediator**	pA238L (71, 73–75)	inhibit p300	inhibit NO
		inhibit TNF-α	inhibit prostaglandin
**ALR**	pS183L、pE199L、pO61R、pI7L (71, 75)	/	Activate AIM2 inflammasome
	pI226L、pA151R、pNP419、pQP383R (75)	/	inhibit AIM2 inflammasome
**NF-κB**	pA238L (71, 73, 74)	inhibit p300/CBP	inhibit p65 acetylation and NF-κB
		binding with p65	inhibit the nuclear transport of NF-κB
		promoting p65 nuclear exporting	
	MGF-360-12L protein (76)	inhibit the interaction between p65 and importin α	
		inhibit the interaction between NF-κB andnuclear transport proteins	

### cGAS/STING Pathway Inhibition

Several PRRs present in host cells identify pathogenic microorganisms intruding into the cells by recognizing the PAMPs. Host cells initiate natural immune responses by recognizing PAMPs *via* PRRs. PRRs include TLRs, NLRs, CLRs, RLRs, and cGAS, while PAMPs include endotoxin, peptidoglycan, dsDNA, dsRNA, ssRNA, ssDNA, and unmethylated DNA. After ASFV infects porcine macrophages, the main PRRs are cGAS to recognize the PAMPs, dsDNA, of the virus. cGAS is a cyclic GMP–AMP synthase located in the cytoplasm, which catalyzes the synthesis of cyclic GMP–AMP (cGAMP) after recognizing the viral dsDNA. Subsequently, cGAMP stimulation induces the type I IFN production and activates the transcription factor NF-κB through the STING–TBK1–IRF3 signaling pathway. IFNs and NF-κB induce antiviral protein synthesis and proinflammatory factor production through downstream pathways to eliminate the virus.

DP96R of the ASFV China 2018/1 strain is a conservative early expression protein that inhibits the phosphorylation of TBK1 induced by cGAS/STING. DP96R inhibits the activation of IFN-β and ISRE promoters by suppressing cGAS/STING and TBK1, but does not inhibit their activation mediated by IRF3-5D. In addition, DP96R can also inhibit the NF-κB promoter by inhibiting cGAS/STING, TBK1, and IKKβ. The C-side 30-96 aa of DP96R is the active site of its inhibition function (14). Additionally, MGF-505-7R interacted with STING in cells infected with ASFV. MGF-505-7R overexpression promotes autophagy-related protein expression, reduces STING expression, and inhibits the cGAS/STING signaling pathway (15). In addition, Tol-like receptor (including TLR1, TLR2, TLR4, and TLR6) expression decreases in ASFV-infected macrophages (16).

### Cytokine Expression Regulation

Cytokines are active protein molecules secreted by cells that perform signal transduction, participate in immune response and inflammation in different ways, and play an important role in maintaining the normal function of the organism and removing pathogenic microorganisms. Cytokines include interleukins, interferons, TNFs, and chemokines. During ASFV infection, 20 cytokines are significantly upregulated in host cells and four cytokines are downregulated (16). Therefore, it is speculated that ASFV regulates the immune response by manipulating cytokine expression in host cells.

#### Interferon Induction and Downstream Gene Expression Inhibition

Interferons are the first line of defense against viral infections and play a considerable role in the early immune response. Type I interferon (IFN-α/β) is an important component of innate immunity, and type II interferon (IFN-γ) is a momentous immune response molecule that participates in the entire process of immune response. Furthermore, the combination of interferon with its receptors activates JAK/STAT signal transduction, inducing the expression of several antiviral proteins related to antigen delivery and apoptosis. Therefore, interferon induction suppression seriously disturbs the antiviral reaction of the host.

The ASFV A276R, derived from the MGF360, targets IRF3 instead of IRF7 and IFN-β through the cytosolic pathway and TLR3 pathway to suppress IFN-β induction. ASFV A528R inhibits IFN-β and IRF3 promoter activation in the type I IFN signaling pathway to inhibit type I IFN generation (15, 77). ASFV I329L is considered to be a TLR3 homolog because it harbors an extracellular leucine sequence similar to that in TLR3 (78). I329L inhibits activation induction of IFN-β, NF-B and IRF3 at the TRIF level (77) and the subsequent expression of IFN-β and CCL5 (79). Moreover, MGF360 and MGF505/530 inhibit the I-type IFN production and impact (14). ASFV MGF360-12L reduces IFN-β and NF-κB promoters activity and inhibits mRNA transcription of IFN-β, IRF3, STING, TBK1, ISG54, ISG56 and AP-1 (76).

#### TNF-α Inhibition

pA238L inhibits TNF-α generation and expression. CBP/p300 is a histone acetyltransferase (HAT) that enables histone acetylation to promote transcription factor-mediated target gene transcription. pA238L replaces the combination of cyclic AMP-responsive element/κ3complex with CBP/p300 on the TNF-α promoter, inhibiting TNF-α activation. In addition, pA238L also inhibits TNF-promoter activation by interfering with the function of CBP/p300 by trans-activating transcription factors NF-κB, NF-AT, and c-Jun (80). The expression of two signal sensors (FADD and TRADD) of TNF were also decreased 9 h post ASFV infection (16). TNF-α is a proinflammatory cytokine that triggers an inflammatory response and induces apoptosis through downstream TNF-α signal transduction. In summary, TNF-α generation and expression inhibition is also a mechanism of ASFV immune evasion and immunosuppression.

#### Interleukin Regulation

Studies have found that ASFV upregulates immunosuppressive cytokine expression to restrain the host immune response. Interleukin-1 (IL-1), an endogenous pyrogen, causes an inflammatory response and promotes B cell proliferation and differentiation when combined with IL-1 receptor (IL-1R). In ASFV-infected cells, IL-1R antagonist (IL-1RN), a natural IL-1 antagonist, increases significantly and inhibits the combination of IL-1 with IL-1R, which competitively inhibits the effect of IL-1. In contrast, the expression of IL27, which suppresses the immune response of Th1 and Th17 cells, is downregulated. ASFV L83L encodes a protein that binds to IL-1β. However, deletion of this gene does not attenuate ASFV, indicating a redundancy mechanism of ASFV that interferes with IL-1 production (16). Till date, the exact mechanism through which ASFV evades the immune response by regulating host cytokines remains unclear.

#### Chemokine Regulation

Chemokines are a class of small proteins that manipulate lymphocyte migration to local inflammation, which assists immune response. ASFV infection significantly induces the chemokine expression to recruit monocytes, T-cells, and natural killer (NK) cells, which are cell-generated antiviral reactions. However, 3 h post ASFV infection, the expression of chemokines, including CXCL1, CXCL2, CXCL3, and CXCL14, responsible for neutral granulocyte and CD8 T-cell recruitment, was inhibited (16).

### Interference With Adaptive Immunity

The 28-kDa form of A238Lp interacts with cyclophilin and the small subunit of calcineurin, blocking NF-AT activation, thus inhibiting T-cell proliferation and differentiation (17). Furthermore, ASFV EP153R regulates MHC-I expression by interacting with MHC-I through NKG2D and ULBP3, which inhibits MHC-I transport from the ER to the cell membrane instead of affecting MHC-I synthesis or maturation (14). MHC-I decrease may inhibit NK cell activation (81, 82). In addition to inhibiting MHC-I, ASFV also prevents the binding of short antigen peptides to MHC-II. MHC-II-coded DMA/DMB removes the class II-associated invariant chain peptide CLIP/CD74 in the groove to promote the binding of exogenous antigen peptides with MHC-II, while MHC-II-encoded DOA/DOB can inhibit this function. About 9 h post ASFV infection, SLA-DMA and SLA-DMB expression reduced in swine macrophages, but SLA-DOA and SLA-DOB expression increased. Thus, ASFV has been suggested to inhibit the immune response by preventing antigen binding to MHC-II. In addition, antigens are digested and processed into short peptides before being loaded onto MHC-II mainly by cathepsins. Cathepsin expression in ASFV-infected cells is lower than that in uninfected cells, which inhibits antigen binding to MHC-II. Therefore, ASFV achieves immunosuppression by inhibiting the processing of MHC-II antigens (16). In addition, CD2v inhibits lymphocyte activation *in vitro* (14).

### Apoptosis Inhibition in Host Cells

There are two common apoptotic pathways: the extrinsic apoptotic pathway (EAP), also known as the death receptor pathway, and the intrinsic apoptotic pathway (IAP), also called the mitochondrial apoptotic pathway. EAP-mediated apoptosis involves TNF family members and death receptors (including FASL/FASR and TNF-α/TNFR1) in the cell membrane. The binding of the death receptor to the ligand produces intracellular apoptosis signals, which then recruits FADD and TRADD as well as caspase 8 to form a death-inducing signaling complex (DISC) and finally activates caspase 8 and caspase 3 successively for apoptosis. However, in IAP-mediated apoptosis, in-cell apoptosis stimulators (e.g., viral proteins) interact with Bcl-2 family apoptosis factors (e.g., Bax, Bak) or anti-apoptotic molecules (e.g., Bcl-2) on the mitochondria, releasing Cyt-c from the mitochondria to the cytoplasm. Cyt-c, APAF-1, and caspase 9 form apoptosomes, which activates caspases 9/3/6/7, thus causing apoptosis. By inducing apoptosis, the host can expose the virus to the immune system and promote virus removal, which is beneficial for maintaining its stability. Hence, to survive, complete replication cycles, and produces more offspring; viruses have evolved an apoptosis-specific immune escape mechanism.

Studies have shown that ASFV A179L, EP153R, DP71L, and A224L suppress ASFV infection-induced apoptosis (14). A179L inhibits both dead receptor-mediated and mitochondrial apoptosis pathways. A179L sequence is very similar to Bcl-2 sequence and considered to be homologous to Bcl-2. A179L is a pan-apoptosis Bcl-2 binding protein (71, 83), maintaining high affinity with upstream proteins (Bid, Bim, and Puma) and downstream proteins (Bax and Bak) to ensure its extensive apoptosis inhibition. The interaction between A179L and anti-apoptotic proteins is mediated by a domain similar to that of BH3 encoded by A179L (71). Bid is cut by caspase8 or granzyme B produced by cytotoxic T cells in the process of killing target cells to produce tBid–p13, the active form of the bid protein. The BH3 domain-dependent interaction between A179L and tBid–p13 inhibits apoptosis (84). However, other studies have concluded that the BH1 domain in A179L is necessary for the combination of A179L and Bcl-2 (72). pE153R has a C-type lectin domain and inhibits p53-mediated apoptosis and caspase 3 activity. A224L, a member of the inhibitor of IAP-mediated apoptosis family, has a BIR sequence and can inhibit apoptosis under TNF-α induction (71). A224L is involved in multiple ways to suppress apoptosis. It regulates the protease processing of caspase 3 by inhibiting caspase 3 protease activity and interacting with the hydrolytic fragments of caspase 3, thus restraining caspase 3 activity (46). It promotes the transcription of many anti-apoptotic genes such as IAP, A20, Bcl-X, Bcl-2, and the IPA family to suppress apoptosis, by activating NF-κB. In addition, it also activates cFLIP to inhibit caspase 8 activities (71, 85). DP71L inhibits apoptosis by suppressing the unfolded protein response induced by ER stress. DP71L recruits PPI in combination with PPI to dephosphorylate eIF-2α and inhibit ATF4 and its downstream pro-apoptotic transcription factor CHOP that triggers ER stress-mediated apoptosis. Thus, DP71L inhibits apoptosis by inhibiting CHOP expression (44, 71). In addition, the expression of the pro-apoptotic gene GADD45A (growth arrest and DNA-damage-inducible 45 α) was also reduced (16).

### Autophagy Inhibition in Host Cells

Autophagy refers to the biological process of incorporating foreign matters in the cellular autophagosome and cyclic utilization through lysosomal degradation. This process is also known as heterophagy, in which pathogenic microorganisms are incorporated into the autophagosome. Autophagy is important for the elimination of viruses and the maintenance of intracellular environmental stability. Thus, viruses have evolved a series of countermeasures to fight autophagy in cells, which is also an aspect of viral immune escape. ATG2A, ATG9A, ATG101, ATG4B, and ATG7 are autophagy-related genes downregulated in ASFV-infected host cells. However, nuclear protein 1 (NUPR1), which inhibits autophagy, is upregulated during ASFV infection. In addition, BCL2 interacting protein 3 (BNIP3) also induces autophagy and apoptosis and is significantly reduced 9 h after ASFV infection. It has been speculated that the combination of the A179L and BH3 domains of Beclin-1 may inhibit autophagy (71). Grooves in the binding region of the A179L spatial configuration are necessary for the combination of A179L and Beclin-1. The ability of the A179L mutant to bind to Beclin-1 decreased the ability of A179L to inhibit autophagy (86).

### Inflammation Regulation

Inflammatory reactions are effective as host antiviral therapies. The host regulates inflammatory responses through proinflammatory factors, inflammatory mediators, and proteins produced in downstream pathways of the NF-κB and ALR pathways. Once the regulation is unbalanced, an inflammatory factor storm is produced in the organism, causing damage to normal cells. The virus controls cellular inflammation by regulating the production and expression of these substances in favor of viral survival (**Table 1**).

#### Inflammatory Mediator Inhibition

Viral proteins inhibit inflammatory reactions by reducing the production of inflammatory mediators, such as NO and prostaglandin. pA238L is an effective anti-inflammatory protein that inhibits the inflammatory response of cells. Inducible nitric oxide synthase (iNOS) induces nitric oxide synthase (NOS) to produce NO, and cyclo-oxygenase (COX) catalyzes arachidonic acid conversion into prostaglandin. iNOS transcription is regulated by the co-activation factor, CBP/p300. P300 overexpression enhances iNOS promoter activity. However, A238L expression indirectly inhibits the activity of iNOS promoters by inhibiting the effect of p300. Moreover, A238L also inhibits TNF-α activation-induced NOS and COX activation pathway, as well as cox-2 transcription and prostaglandin production.

#### NF-κB Pathway Inhibition

In addition to suppressing inflammation by inhibiting NO, prostaglandin, and NOS production, pA238L inhibits inflammation by inhibiting NF-κB. First, A238L suppresses NF-κB by inhibiting acetylation of p65. p300/CBP is a histone acetyltransferase (HAT) enzyme, which directly acetylates p65. A238L prevents p300/CBP-mediated p65 acetylation, thus reducing its affinity for DNA binding (71, 73, 74). Secondly, A238L, an IκBα homolog, has an Ankyrin repeat sequence similar to IκBα. The translation-modified 32-kDa form of A238L directly interacts with the 65 kDa subunit of NF-B to form the A238L–p65 complex located in the cytoplasm, inhibiting p65 entry in the nucleus and the binding of NF-κB to DNA. In addition, unlike IκBα, A238L also inhibits NF-κB activity by enhancing NF-κB export in the nucleus. NF-κB activity inhibition suppresses of the expression of inflammatory cytokines initiated through the NF-κB signal transduction pathway (17). Besides, MGF360-12L inhibits NLS-mediated intranuclear NF-κB localization. Karyopherin, comprising importins α and β, is responsible for NF-κB localization. The interactions of MGF360-12L with KPNA2 (importin α2), KPNA3 (importin α3), and KPNA4 (importin α4) inhibit the interaction between p65 and importin α. Moreover, MGF360-12L inhibits the nuclear transport of NF-κB by inhibiting the interaction between NF-κB and nuclear transport proteins (76).

However, some studies have reported opposite results. Rhiannon et al. have found that A238L accumulates in the nucleus during the late-stage infection of recombinant ASFV strains. Moreover, A238L expression neither inhibits the nuclear import of NF-κB p50 or p65 subunit, nor inhibits the CRM1-mediated nuclear exit of p65 (73).

Interestingly, A238L is an early protein that inhibits the inflammatory response of cells, while A224L is a late protein in the viral cycle that promotes it. This may indicate that the virus needs low NF-κB activity at an early stage to avoid an immune response, but high activity at a later stage may prevent apoptosis (85).

#### ALR Pathway and Inflammation Regulation

ASFV also regulates cellular inflammation by controlling the ALR pathway. AIM2 inflammasomes are produced after ALR recognizing the dsDNA located in the cytoplasm. Interestingly, four ASFV-encoded proteins, S183L, E199L, O61R, and I7L, activate the AIM2 inflammasome in the ASFV-infected host cell, while four ASFV-encoded proteins, I226L, A151R, NP419L, and QP383R, inhibit AIM2 inflammasome activation (75).

In addition, the combination of pL83L and IL-1β may regulate inflammatory factor activity (71).

## Discussion

Till date, there are no effective vaccines against ASFV, which makes ASF prevention or control difficult. Therefore, basic research on ASFV is still needed to provide a theoretical basis for vaccine research. On the one hand, the life cycle of ASFV in host cells needs to be further studied. On the other hand, the host immune response to ASFV and the immunosuppressive mechanism of ASFV evolution need a further research.

Although the detailed process of ASFV infected cells has been described in previous studies, the involved proteins of cells and viral are still unknown. It is possible that multiple molecules as receptors or co-receptors are involved in the viral infection process. The viral proteins involved in entry into host cells can be used as targets for ASFV attenuation, which offers possibilities for successful vaccine development. When ASFV successfully enters host cells, it forms a VF near the perinuclear region, which is necessary for newborn virus assembly. VF is a type of membrane; hence VF formation requires lipid accumulation. However, the mechanism of VF membrane structure formation is unclear. Some studies have suggested that VF originates from the ER and intracellular vesicular system, while others have observed viral protein expression collapses the ER (5, 61). In addition, ASFV also replicates within the nucleus, and ASFV DNA entry into the nucleus may be related to viral proteins p37, p14, and p10 that can enter the nucleus (7, 49–51). However, the viral protein-mediated transport of large viral DNA to the nucleus, DNA replication, and DNA transport to cytoplasm should be studied in the future.

As is known to all, the organism produces a series of antiviral responses due to the invasion of ASFV, while ASFV can also form its own immune escape mechanism against the antiviral responses. Aiming at the production of type I interferon through cGAS-STING signal pathway, virulent ASFV strain can inhibit the production of type I IFN by regulating some adaptor molecules of this signal pathway, such as regulating the activity of IRF3, the phosphorylation of STING and its translocation in cytoplasm. As for the antiviral effect of macrophages and dendritic cells, ASFV can down regulate the expression of CD14 and CD16 in infected macrophages, which weaken its antiviral and antimicrobial response. Compared with attenuated strain, virulent ASFV strain cannot activate NK cells by down regulating the expression of MHCI in macrophages and dendritic cells, thereby avoiding the recognition of host innate immunity. In addition, the virulent strain may also have the ability, lost by attenuated strain, to avoid the production and release of key cytokines (IFN-α, IFN-β, IL-1β and so on) by macrophages, which weakens the immune surveillance and T cell response ability of macrophages. In addition, some ASFV genes can also inhibit transcription factors (such as IRF3 and NF-κB) to inhibit the production of type I IFN in macrophages, such as ASFV multigene families (MGF) 360 and 530/505 and I329l. Virulent ASFV strain can also inhibit the expression of ISGS. As for the adaptive immunity produced by host cells, ASFV may inhibit the immune response through regulatory T cells (70).

However, ASFV genome encodes more than 100 non-structural proteins, most of which are involved in immune response regulation. The mechanisms for immune invasion and immunosuppression by ASFV have been partially elucidated, but it mostly remains unclear. ASFV often inhibits the expression of certain antiviral proteins or immune responses in several ways. For example, A276R and I329L inhibit IFN-β expression through different mechanisms, and A179L, A224L, and DP71L inhibit apoptosis. This redundancy may also be one reason for the difficulty in developing vaccines. Therefore, studies on the functions of ASFV-encoded proteins, particularly the proteins mediating ASFV immune escape and immunosuppression, is very important for a live-attenuated vaccine development and design.

In summary, the next work will focus on a comprehensive study and analysis of the function of ASFV protein. We will focus on the proteins involved in the virus life cycle (adsorption, internalization, shelling, biosynthesis, assembly and release) and immunosuppressive function. Because these proteins are most likely the key virulence factors of virus, they are very important for the development of attenuated vaccine. Weakening the virulence gene of virus is one of the strategies to construct attenuated vaccine. For example, the attenuated vaccine of Fowlpox virus was obtained by weakening (87, 88). Similarly, an effective vaccine for Ebola virus is developed by replacing the glycoprotein on the surface of recombinant Vesicular Stomatitis Virus with the glycoprotein that can recognize Ebola virus (89, 90).

## Author Contributions

YW, HZ and DL conceived and designed the study. YW, WY, WK, JZ, HZ, and DL wrote the manuscript. All authors contributed to the article and approved the submitted version.

## Funding

This work was supported by grants from the Gansu major science and technology projects (20ZD7NA006), National Natural Science Foundation of China (31941002).

## Conflict of Interest

The authors declare that the research was conducted in the absence of any commercial or financial relationships that could be construed as a potential conflict of interest.

## Publisher’s Note

All claims expressed in this article are solely those of the authors and do not necessarily represent those of their affiliated organizations, or those of the publisher, the editors and the reviewers. Any product that may be evaluated in this article, or claim that may be made by its manufacturer, is not guaranteed or endorsed by the publisher.
